# Potential of seaweed (*Eucheuma cottonii*) supplementation to reduce methane production, improve fermentation, and modulate the microbial composition of forages and crop by-products during *in vitro* rumen fermentation

**DOI:** 10.3389/fvets.2025.1607879

**Published:** 2025-09-22

**Authors:** Yeni Widiawati, Slamet Widodo, Moyosore Joseph Adegbeye, Ferdy Saputra, Edwin Rafael Alvarado-Ramírez, Nadia Alejandra Sánchez Guerra, Yenny Nur Anggraeny, Wisri Puastuti, Dwi Yulistiani, Eni Siti Rohaeni, Eko Handiwirawan, Lisa Praharani

**Affiliations:** ^1^Research Center for Animal Husbandry, National Research and Innovation Agency (BRIN), Bogor, Indonesia; ^2^Faculty of Veterinary Medicine and Zootechnics, Autonomous University of Tamaulipas, Victoria City, Tamaulipas, Mexico

**Keywords:** crop by-products, *Eucheuma cottonii*, forages, greenhouse gases, *in vitro* fermentation, ruminal microbial diversity, ruminants, seaweed

## Abstract

**Introduction:**

In Indonesia, small-scale livestock farmers commonly feed their animals with forage resources that are high in fiber and low in digestibility, which contributes to increased methane (CH_4_) production in ruminants. To address this issue, algae, including marine species, have shown significant potential to improve rumen fermentation profiles, modulate microbial composition, and reduce CH_4_ emissions in these animals. Therefore, the aim of the present study was to evaluate the impact of supplementation with the marine seaweed *Eucheuma cottonii* on total gas and CH_4_ production, degradability, fermentation end-products, and rumen microbial composition in forages and crop by-products, using an *in vitro* system.

**Methods:**

The forages and crop by-products evaluated were buffel grass (GB; *Cenchrus ciliaris*), elephant grass (EG; *Pennisetum purpureum*), rice straw (RS), corn stalk (CS), oil palm leaves (PL), and sugarcane leaves (SC). Seaweed supplementation was carried out by replacing a proportion of the dry matter (DM) in the basal diet corresponding to each forage or crop by-product, with inclusion levels of 0, 4, 8, and 12%, calculated on a DM basis. The forages and crop by-products were obtained from local farms in Serang and represent the most commonly used basal feed sources for ruminants by small-scale farmers and industry stakeholders. Seaweed was collected during the dry season, in July 2021, 45 days after planting, from a cultivation site located in Serang, Banten, Indonesia.

**Results and discussion:**

Result showed that corn stalk produced the highest asymptotic gas, dry matter digestibility, shorted fermentation lag time, SCFA, metabolizable energy, and microbial crude protein. Corn stalk production the highest asymptotic (*p* < 0.001) methane gas, but had the lowest proportion of methane gas compared to total gas. Microbial analysis showed that rice straw without seaweed had the highest microbial diversity and evenness while rice straw with 8% seaweed group, exhibited lower methanogen abundance, increased *Rikenellaceae_RC9* gut group and *Ruminobacter*. Cornstalk was the most efficient forage in rumen fermentation, while *E. cottonii* supplementation modulated fermentation, enhanced microbial protein synthesis, reduced methane emissions, and altered microbial diversity. Therefore, corn stalks without seaweed is a highly effective crop-by product for ruminant nutrition offering better fermentation characteristic and energy yield.

## 1 Introduction

Smallholder farmers, who practice mixed crop and livestock systems throughout the year, and pastoralists, who adjust livestock feeding according to the season, largely depend on crop by-products as their main source of feed due to their availability and low cost (1), which represents an efficient resource utilization strategy and contributes to mitigating environmental pollution by reducing waste accumulation and preventing open burning. In Indonesia, the main crop by-products used in livestock feeding include rice straw, corn stalks, oil palm leaves, and sugarcane leaves (2), although in some regions, buffel grass (*Cenchrus ciliaris*) and elephant grass (*Pennisetum purpureum*) are also used as primary forage sources. Ruminant livestock, in their role as “biological transformers,” play a key role in the utilization of fibrous resources of low nutritional value by converting them into high-value products for human consumption, such as meat and milk, which provide high-quality proteins, essential amino acids, and fatty acids (3). This capacity is due to the abundant and diverse microbial community present in the rumen, composed of bacteria, protozoa, fungi, archaea, and bacteriophages, and to a lesser extent in the large intestine, which enables the efficient degradation of structural and non-structural polysaccharides found in forages and crop by-products not suitable for human consumption (4).

Although the use of forages and crop by-products in ruminant feeding allows for efficient resource utilization and offers environmental benefits (3), this approach cannot be considered fully sustainable due to methane (CH_4_) emissions derived from ruminal fermentation, a potent greenhouse gas (GHG). While CH_4_ contributes to regulating hydrogen levels in the rumen, preventing fermentation inhibition, it also represents energy and carbon losses that, under ideal conditions, could be directed toward the synthesis of short-chain fatty acids, which are essential nutrients for livestock (5). Moreover, CH_4_ production tends to increase with the fermentation of high-fiber diets, as these types of substrates stimulate the activity of methanogenic microorganisms in the rumen (6). In Indonesia, ruminant livestock, particularly cattle, constitutes a significant source of CH_4_ through enteric fermentation, contributing substantially to GHG emissions (7). According to estimates based on a Tier 2 approach, GHG emissions from livestock in Indonesia reached 30.05 gigagrams of carbon dioxide equivalent (Gg CO_2_-e)/year in 2020, and are projected to reach 59.10 Gg CO_2_-e/year by 2030, representing an annual increase of 9.67%, mainly driven by the expansion of the cattle population (8). Furthermore, GHG emissions from beef cattle, an important source of meat in the country, are expected to increase from 18.90 Gg CO_2_-e/year in 2020 to 36.96 Gg CO_2_-e/year in 2030 (8). This scenario has prompted the search for strategies to mitigate enteric CH_4_ emissions, including dietary supplementation and the use of feed additives such as essential oils, probiotics, and prebiotics (9). However, despite their effectiveness in inhibiting methanogenic archaea, these additives are often costly or have limited availability, making them difficult to adopt for small-scale farmers with restricted financial resources.

A promising alternative is seaweed, an underutilized natural resource that emerges as a potential tool to improve the sustainability of ruminant livestock systems through its incorporation as a dietary supplement (10). Seaweeds, including green, red, and brown types, are rich in bioactive compounds such as polysaccharides, proteins, essential amino acids, minerals, lipids (including polyunsaturated fatty acids), polyphenols, vitamins, pigments (chlorophylls and carotenoids), and numerous antioxidants, which can improve the quality of the basal diet (11). In addition, due to their antibacterial, antifungal, antiviral, antioxidant, and anti-inflammatory properties, seaweeds can not only enhance animal production but also contribute to the sustainability of livestock systems (12). In particular, red and brown seaweeds have demonstrated in *in vitro* studies their ability to reduce CH_4_ production without compromising degradability, by favorably modulating the structure of the ruminal microbial community, especially through the inhibition of methanogenic archaea, the microorganisms responsible for CH_4_ production (6, 13), which is associated with greater fermentation efficiency and improved animal growth performance (12). Among them, *Eucheuma cottonii*, a red seaweed of high commercial and ecological value due to its ability to sequester CO_2_, is mainly cultivated in countries in Asia and the Pacific, as well as in Africa and Brazil (14). It is rich in carrageenan, a sulfated polysaccharide that has been shown to improve digestibility and reduce GHG emissions in ruminants (15, 16), which translates into greater production of short-chain fatty acids and availability of metabolizable energy for animal growth (17). Based on this evidence, there is growing interest in evaluating the supplementation of *E. cottonii* in high-fiber diets, such as those based on forages and crop by-products commonly used by small-scale farmers in Indonesia. However, the potential of seaweeds to reduce ruminal CH_4_ production largely depends on the type of forage, its chemical composition, and the level of inclusion or supplementation (4, 17), suggesting that the use of *E. cottonii* as a supplement in forages and crop by-products could produce variable responses in CH_4_ mitigation and alter the ruminal fermentation profile. Therefore, the objective of the present study was to evaluate the impact of supplementation with different levels of the seaweed *E. cottonii* on total gas and CH_4_ production, degradability, fermentation end-products, and ruminal microbial composition in forages and crop by-products using an *in vitro* system.

## 2 Materials and methods

### 2.1 Experimental treatments

**Figure 1:**
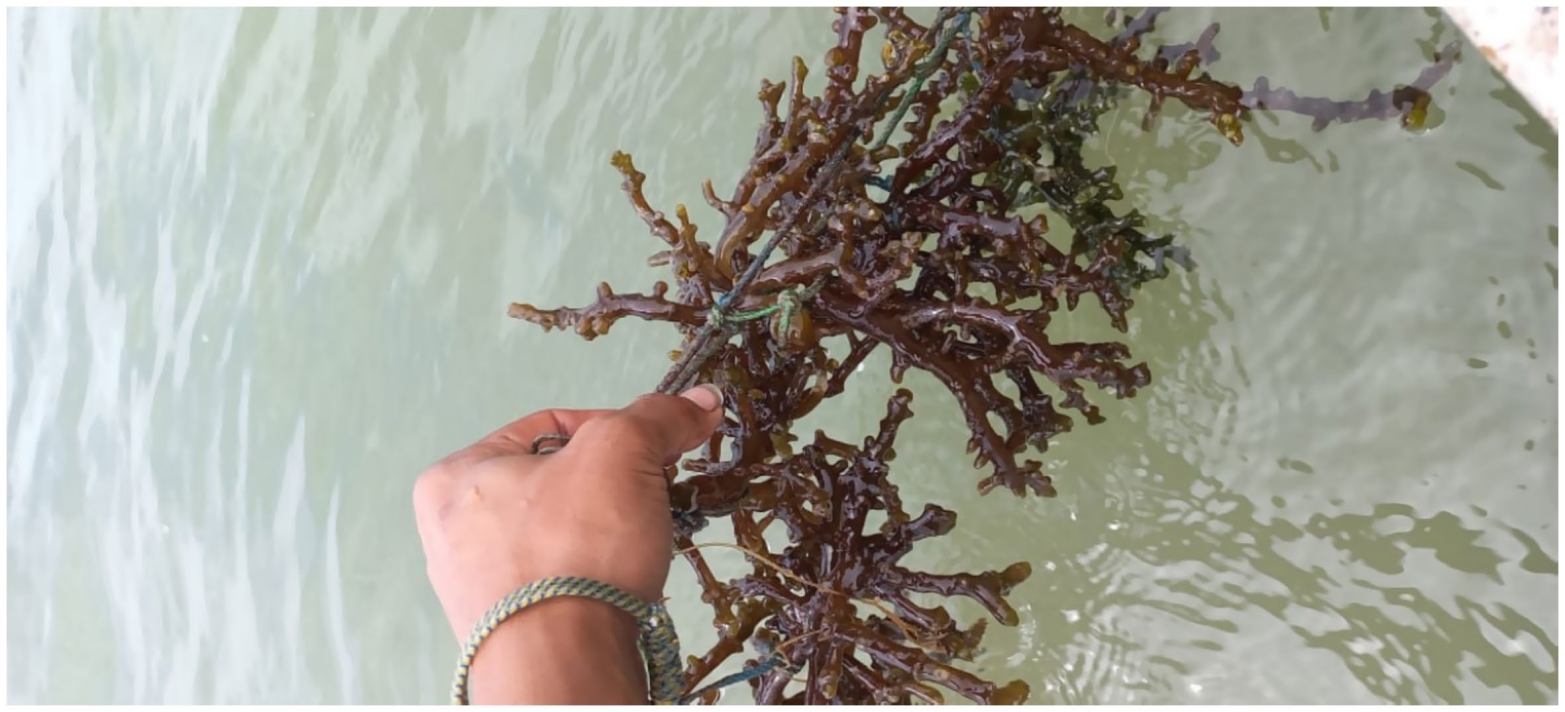
General appearance of *Eucheuma cottonii* used as a supplement in this study.

The factors evaluated in the present study were two: the type of forage or crop by-product, and the supplementation with the seaweed *Eucheuma cottonii*, which will hereafter be referred to simply as “seaweed,” since only one species was evaluated. The forages included buffel grass (BG; *Cenchrus ciliaris*) and elephant grass (EG; *Pennisetum purpureum*), while the crop by-products evaluated were rice straw (RS), corn stover (CS), oil palm leaves (PL), and sugarcane leaves (SC). The supplementation with seaweed was carried out by replacing a proportion of the dry matter (DM) of the basal diet corresponding to each forage or crop by-product, with inclusion levels of 0, 4, 8, and 12%, calculated on a DM basis. Consequently, four treatments were established for each type of forage or crop by-product: T0 (control), consisting of 100% basal diet; T1, with 96% basal diet + 4% seaweed; T2, with 92% basal diet + 8% seaweed; and T3, with 88% basal diet + 12% seaweed. The two forages and four crop by-products used in this study, obtained from local farms, represent the most employed basal diet sources for ruminant feeding by small-scale farmers and industry stakeholders, and were subjected to drying in a forced-air oven at 65°C for 48 h. The seaweed was harvested during the dry season, in July 2021, 45 days after planting, at a cultivation site located in Tirtayasa, a subdistrict of Serang Regency, Banten Province, Indonesia (5°58′16.7” S, 106°18′27.4″E). After collection, the samples underwent a cleaning process consisting of thorough washing with fresh water to remove potential impurities, followed by drying at ambient temperature for 48 h and subsequently by additional drying in a forced-air circulation oven at 65°C for another 48 h. The forages, crop by-products, and seaweed were ground using a hammer mill with a 1 mm screen and stored in vacuum-sealed plastic bags at room temperature for subsequent analysis and *in vitro* experimentation. Figure 1 shows the seaweed species used in this study, which was morphologically identified as *Eucheuma cottonii* by local farmers involved in its cultivation under a government program in Indonesia, with its identity subsequently confirmed by comparing its characteristics to descriptions found in the scientific literature.

### 2.2 Chemical analysis

The chemical composition, as well as the calcium, phosphorus, and energy contents, were analyzed in the two forage samples, the four crop by-products, and the seaweed prior to their use in the *in vitro* experiment (Table 1). The determination of DM, organic matter, and ether extract (EE) was carried out according to the method described by Horwitz and Latimer (18). Neutral detergent fiber (NDF) and acid detergent fiber (ADF) were analyzed following the procedure of Van Soest et al. (19), using *α*-amylase for NDF sample treatment and a neutral detergent solution containing sodium sulfite, and both values were expressed without correction for residual ash. Crude protein content was determined using the Kjeldahl method by multiplying the nitrogen content by a factor of 6.25, while mineral content was analyzed by atomic absorption spectroscopy (AAS), and energy content was estimated using a bomb calorimeter, following the procedures described by Association of Official Analytical Chemists (20).

**Table 1:** Chemical composition and calcium and phosphorus content of forages, crop by-products, and the seaweed *Eucheuma cottonii*.

Item ^1^	Buffel grass	Elephant grass	Corn stalk	Oil palm leaves	Rice straw	Sugarcane leaves	Seaweed *Eucheuma cottonii*
DM (g kg^−1^ FM)	913.0	897.0	857.0	881.0	884.0	903.0	885.1
OM (g kg^−1^ DM)	875.0	899.0	919.0	835.0	808.0	935.0	599.2
PC (g kg^−1^ DM)	80.40	90.0	55.0	84.0	66.0	71.0	84.2
EE (g kg^−1^ DM)	30.0	12.0	10.0	29.0	11.0	19.0	7.4
CF (g kg^−1^ DM)	248	375	288	300	265	398	60.2
NDF (g kg^−1^ DM)	573.0	614.0	588.0	613.0	504.0	723.0	116.9
ADF (g kg^−1^ DM)	487.0	477.0	554.0	500.0	449.0	477.0	117.7
ADL (g kg^−1^ DM)	68	58	56	122	48	56	-
Ca (g kg^−1^ DM)	15.0	10.0	7.0	14.0	10.0	8.0	0.5
P (g kg^−1^ DM)	4.0	3.0	2.0	1.0	1.0	1.0	0.5
Energy (MJ kg^−1^)	15.94	15.61	14.97	15.07	13.00	16.55	9.18

^1^ FM, fresh matter; DM, dry matter; OM, organic matter; CP, crude protein; EE, ether extract; CF, crude fiber; NDF, neutral detergent fiber; ADF, acid detergent fiber; ADL, acid detergent lignin; Ca, calcium; P, phosphorus.

#### 2.2.1 Qualitative analysis of phytochemical compounds in test ingredients

Three replicates of the supernatant obtained from the aqueous extracts were qualitatively analyzed for phytochemical constituents, including alkaloids, flavonoids, saponins, steroids, and tannins, following the methods described by Harborne (21) (Table 2).

**Table 2:** Qualitative analysis of Phytochemical compounds in test ingredients.

Material	Alkaloids	Flavonoids	Saponins	Sterioids	Tannins
Buffel grass	+	++	+	−	+
Elephant grass	++	++	+	−	++
Corn stalk	−	+	+	−	−
Oil palm leaves	++	+	+++	+	+
Rice straw	+	+	++	−	+
Sugar cane leaves	+	++	++	+	++
*E. cottonii*	+++	+	+	−	−

(−) = absent; (+) = present less dense; (++) = present dense; (+++) = present more dense.

### 2.3 *In vitro* incubation

The nutrient medium was prepared according to the methodology described by Goering and Van Soest (22), using analytical grade reagents and including buffer solution, macro-minerals, micro-minerals, a reducing agent, and resazurin. Rumen content was collected in the morning, before feeding, from two fistulated cattle (350 ± 25 kg body weight) and was immediately placed in a thermos previously preheated to 39 ± 1°C and purged with carbon dioxide (CO_2_), in order to avoid thermal shock and preserve anaerobic conditions during its transfer to the laboratory, which was completed within no more than 30 min. Once in the laboratory, the content was filtered through four layers of cheesecloth to remove solid particles and was maintained at 39 ± 1°C under a continuous flow of CO_2_, in order to preserve an anaerobic environment (23). The incubation was carried out in 125 mL bottles purged with CO_2_, in quintuplicate, by adding 500 mg of sample (forage or crop by-product), the corresponding levels of seaweed (0, 4, 8 y 12%, on DM basis), 40 mL of nutrient medium, and 10 mL of rumen fluid, maintaining a 4:1 (v/v) ratio. The bottles were sealed with butyl rubber stoppers and aluminum crimps, gently shaken, and placed in a water bath at 39°C for 48 h.

### 2.4 Measurement of gas production

Total gas (TG) production was measured in PSI at 2, 4, 8, 12, 18, 24, 36, and 48 h of incubation, directly in the bottles, using a digital manometer and following the methodology described by Theodorou et al. (24). Methane (CH_4_) production was determined by extracting a known-volume aliquot from each bottle at the same sampling times, which was injected into a gas chromatograph (Shimadzu GC-8A, Shimadzu Corporation, Kyoto, Japan) equipped with a stainless-steel column packed with activated carbon (1 mm inner diameter × 1 m length). The carrier gas was nitrogen at a flow rate of 50 mL/min, and the temperatures of the injector, column oven, and flame ionization detector were set at 190, 150, and 190°C, respectively. After each measurement, the gas accumulated in the headspace of the bottles was released to prevent partial dissolution of gases and potential errors in the estimations.

### 2.5 Degradability and fermentation end-products

At the time of the final gas measurement (at 48 h), the bottles were opened, and their contents were filtered using 50 mL Gooch crucibles (Pyrex™ brand) with sintered glass discs and a porosity of 40 to 60 μm. The residual material retained in the crucibles was dried in a forced-air oven at 105°C for 24 h, after which the individual weight of each crucible was recorded to determine DM degradability. Subsequently, the contents were ashed in a muffle furnace at 525°C for 4 h, the crucibles were weighed again, and the values obtained were used to estimate organic matter degradability, following the formulas described by Limas-Martínez et al. (23).

Immediately after filtering the contents of the bottles, a 1 mL aliquot was taken from each, which was then mixed with 200 μL of 25% metaphosphoric acid in 1.5 mL Eppendorf tubes and stored at 4°C until analysis. These samples were used to determine the major short-chain fatty acids (acetic, propionic, and butyric), as well as the branched-chain short-chain fatty acids (isobutyric and isovaleric) and valeric acid, in addition to ammoniacal nitrogen, as fermentation end-products. Short-chain fatty acids were analyzed according to the method described by Sondakh et al. (25), using a gas chromatograph (Shimadzu GC-8A, Shimadzu Corporation, Kyoto, Japan) equipped with a glass column containing a stationary phase of FFAP (Free Fatty Acid Phase) and a flame ionization detector. The equipment was operated under isothermal conditions, with the oven temperature set at 130°C; the injector and detector temperatures were set at 200°C, and nitrogen was used as the carrier gas at a flow rate of 40 mL/min. Ammoniacal nitrogen content was determined spectrophotometrically using the phenol-hypochlorite reaction described by Weatherburn (26).

### 2.6 Rumen microbial diversity

In this analysis, only rice straw was evaluated, with and without the inclusion of 8% seaweed, as this by-product is the most commonly used by farmers among those assessed in the present study, and this level of inclusion showed the most promising results. Microbial genomic DNA was extracted from rumen content samples using the Quick-DNA Fecal/Soil Microbe Miniprep Kit D6010 (Zymo Research, USA), following the manufacturer’s instructions. Sequencing of the 16S rRNA gene was performed at NovogeneAIT Genomics (located in Biopolis, Queenstown District, Singapore) using the Illumina MiSeq platform, a high-throughput sequencing system. Primer and barcode trimming, sequence quality control, alpha diversity analysis, and taxonomic assignment were conducted using QIIME2 (27).

### 2.7 Calculations and statistical analyses

The asymptotic production, production rate, and lag time before the onset of TG and CH_4_ production were estimated using the NLIN procedure of the SAS statistical software, version 9.2 (28), following the nonlinear model proposed by France et al. (29):


$$y=b\times [1-e^{-c\;(t-\mathrm{Lag})}]$$

where *y* is the volume (mL) of TG or CH_4_ produced at time *t*; *b* is the asymptotic production of TG or CH_4_ (mL g^−1^ DM); *c* is the production rate of TG or CH_4_ (mL h^−1^); and *Lag* represents the lag time (h) before the onset of TG or CH_4_ production.

Metabolizable energy (ME; MJ kg^−1^ DM) and microbial crude protein (MCP; mg g^−1^ DM) were calculated using the equations proposed by Menke and Steingass (30) and Blümmel et al. (31), respectively:


$$\mathrm{ME}=2.20+(0.136\times \mathrm{TG}P_{24})+(0.057\times \mathrm{CP})$$

$$\mathrm{MCP}=\mathrm{DMD}-(T{\mathrm{GP}}_{24}\times 2.2\;)$$

where *TGP₂₄* is the TG production (mL 200 mg^−1^ DM) at 24 h of incubation; *CP* is the crude protein content (%, on a DM basis); *DMD* is the dry matter degradability (mg g^−1^ DM); and 2.2 is the stoichiometric factor representing the specific carbon, hydrogen, and oxygen requirements (mg) needed to produce 1.0 mL of gas.

Microbial synthesis efficiency was evaluated using the partitioning factor (PF; mg DMD mL^−1^ gas), which was obtained by dividing the DMD (mg) by the volume (mL) of gas production (31). The data were analyzed using a completely randomized design with a factorial arrangement (6 types of forage/crop by-product × 4 levels of seaweed inclusion) and five replicates, using the GLM procedure of SAS version 9.1 (28) with the following statistical model:


$$Y_{\mathrm{ijk}}=\mu +A_{i}+B_{j}+{\left(A\times B\right)}_{\mathrm{ij}}+\varepsilon_{\mathrm{ijk}}$$

where *Y_ijk_* is the response variable; *μ* is the overall mean; *A_i_* is the effect of the type of forage or crop by-product; *B_j_* is the effect of the level of seaweed supplementation; *(A × B)_ij_* is the effect of the interaction between the type of forage or crop by-product and the level of seaweed supplementation; and *ε_ijk_* is the experimental error. Linear and quadratic effects of seaweed supplementation levels on forages and crop by-products were evaluated using orthogonal polynomial contrasts. Statistical differences were considered significant at *p* < 0.05 and interpreted as a trend when 0.05 ≤ *p* < 0.10.

## 3 Results

### 3.1 Gas production and methane

**Figure 2:**
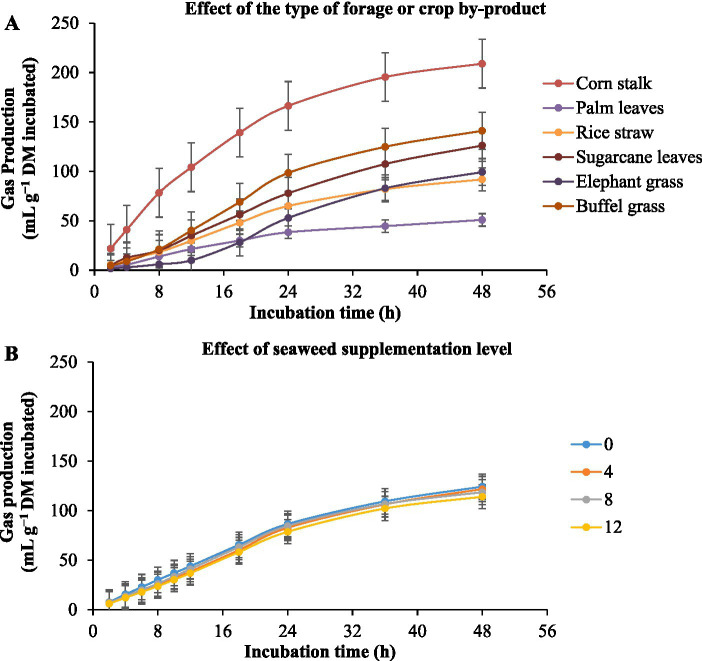
Kinetics of *in vitro* ruminal total gas production in different types of forage or crop by-product [**(A)** corn stalk, palm leaves, rice straw, sugarcane leaves, elephant grass, and buffel grass] with the supplementation of increasing levels of the seaweed *Eucheuma cottonii* [**(B)** 0, 4, 8, and 12%, on a dry matter basis]. The bars at each point represent the standard error of the mean.

The *in vitro* ruminal gas production of different substrates with *E. cottonii* seaweed supplementation is given in Table 3 and Figure 2. Figure 2 illustrates the gas production (mL/g DM incubated) as influenced by roughage type. The results showed that gas production increased throughout the 48-h incubation period, regardless of the diet, with the corn stalk substrate producing the highest gas volume at 48 h, while palm oil leaves produced the lowest. Furthermore, Seaweed level did not significantly (*p* > 0.05) affect gas production (mL g^−1^ DM incubated). Corn stalk substrate produced the highest asymptotic gas volume (*p* < 0.0001), followed by buffel grass, sugarcane tops, elephant grass, rice straw, and palm oil leaves (Table 3). The lag time analysis revealed that the corn stalk substrate had the fastest (*p* < 0.0001) initiation of gas production, followed by palm oil leaves, rice straw, sugarcane tops, and native grass, while elephant grass exhibited the longest delay before gas production began.

**Table 3:** Parameters and *in vitro* ruminal total gas production in different types of forages and crop by-products supplemented with increasing levels of the seaweed *Eucheuma cottonii*, at 8, 24, and 48 h of fermentation.

Forage and by-product	Seaweed level (%)	Parameters ^1^			Total gas production (mL g^−1^ DM incubated)		
		*b*	*c*	*Lag*	8 h	24 h	48 h
Buffel grass	0	144.74	0.042	0.58	23.49	106.66	149.31
	4	128.44	0.042	0.69	16.74	89.84	132.98
	8	140.46	0.041	0.56	23.59	101.56	143.34
	12	134.46	0.042	0.61	20.41	95.61	138.51
Elephant grass	0	93.41	0.037	1.18	6.48	42.98	91.69
	4	103.25	0.044	1.14	6.22	59.22	106.19
	8	99.30	0.045	1.13	6.90	58.81	101.15
	12	96.63	0.047	1.34	4.18	50.52	98.03
Corn stalk	0	220.84	0.037	0.17	89.52	179.99	228.58
	4	216.36	0.036	0.13	81.34	175.48	221.21
	8	189.54	0.038	0.08	70.78	154.84	193.97
	12	187.30	0.039	0.08	71.49	154.85	192.16
Oil palm leaves	0	56.36	0.101	0.11	16.89	42.82	58.03
	4	46.57	0.044	0.36	11.36	38.07	48.95
	8	50.19	0.046	0.26	14.14	40.43	54.95
	12	40.75	0.038	0.08	12.75	32.25	41.52
Rice straw	0	94.07	0.037	0.30	21.03	68.26	95.09
	4	95.06	0.033	0.40	17.45	61.42	93.29
	8	91.48	0.037	0.28	20.95	70.88	91.59
	12	85.04	0.038	0.51	14.12	59.27	87.41
Sugarcane leaves	0	123.38	0.033	0.43	23.25	78.37	122.85
	4	130.62	0.032	0.65	16.50	72.55	128.20
	8	125.59	0.036	0.44	20.34	80.88	126.81
	12	126.66	0.034	0.53	19.55	79.55	126.65
Pooled SEM ^2^		3.690	0.0140	0.044	1.788	2.963	3.492
*p*-value							
Forage or by-product		<0.0001	0.2186	<0.0001	<0.0001	<0.0001	<0.0001
Seaweed level		<0.0001	0.6539	<0.0001	<0.0001	0.0001	<0.0001
Linear		0.1457	0.3626	0.8030	0.0013	0.7462	0.1557
Quadratic		0.0370	0.3821	<0.0001	<0.0001	0.0001	0.0153
Forage or by-product × Seaweed level		<0.0001	0.6640	<0.0001	<0.0001	<0.0001	<0.0001

b, asymptotic total gas production (mL g^−1^ DM); c, rate of total gas production (h^−1^); Lag, initial delay before total gas production begins (h); ^2^ SEM, standard error of the mean.

Results demonstrated that seaweed level had no significantly effect (*p* > 0.05) effect on asymptotic gas production and the volume of gas produced per gram of dry matter incubated at 24, and 48 h of incubation. Although the differences were not statistically significant (*p* > 0.05), gas production showed a numerical decrease with increasing levels of *Eucheuma cottonii.* Additionally, the substrate with 4% *Eucheuma cottonii* powder exhibited the longest lag time before gas production commenced, while the diet containing 8% *E. cottonii* powder had the shortest lag time.

The interaction between forage type and seaweed inclusion revealed substrate-dependent effects. Corn stalk, palm oil leaves, and buffel grass without seaweed, as well as rice straw, sugarcane tops, and elephant grass containing 4% *E. cottonii* powder, produced the highest (*p* < 0.0001) asymptotic gas volume and gas yield per gram of DM incubated or degraded. Additionally, rice straw, elephant grass, and buffel grass with 8% seaweed powder, corn stalk and palm oil leaves with 12% seaweed powder, and sugarcane tops with 4% seaweed powder exhibited the shortest (*p* < 0.0001) lag times for initial gas production.

**Figure 3:**
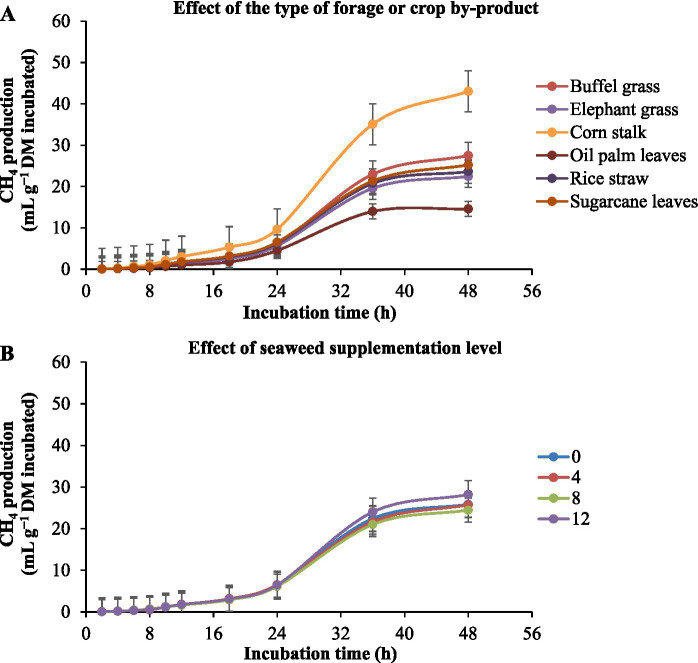
Kinetics of *in vitro* ruminal methane (CH_4_) production in different types of forage or crop by-product [**(A)** corn stalk, palm leaves, rice straw, sugarcane leaves, elephant grass, and buffel grass] with the supplementation of increasing levels of seaweed *Eucheuma cottonii* [**(B)** 0, 4, 8, and 12%, on a dry matter basis]. The bars at each point represent the standard error of the mean.

Methane production during *in vitro* rumen fermentation of different substrates with increasing levels of *E. cottonii* seaweed are shown in Table 4 and Figure 3. The results indicated that corn stalk produced the highest asymptotic methane, followed by elephant grass, while oil palm leaves produced the lowest methane gas (*p* < 0.0001). However, in 24 h of incubation corn stalk had the lowest proportion of methane from the total gas while oil palm leaves had the highest (*p* < 0.05). The effect of seaweed supplementation showed that substrates with 8% seaweed produced the lowest methane and proportion of methane in the total gas (*p* < 0.01) The interaction between substrate type and seaweed level (*p* < 0.05) showed that methane production was lowest in corn stalk and buffel grass without seaweed, elephant grass with 12% seaweed, palm oil leaves, rice straw, and sugarcane tops with 8% seaweed.

**Table 4:** Parameters and *in vitro* ruminal methane (CH_4_) production in different types of forages and crop by-products supplemented with increasing levels of the seaweed *Eucheuma cottonii*, at 8, 24, and 48 h of fermentation.

Forage and by-product	Seaweed level (%)	Parameters ^1^			CH_4_ production (mL g^−1^ DM incubated)			CH_4_ proportion ^2^ (mL 100 mL^−1^ TG)		
		*b*	*c*	*Lag*	8 h	24 h	48 h	8 h	24 h	48 h
Buffel grass	0	23.79	0.053	1.92	0.52	5.78	23.11	8.20	14.00	25.34
	4	21.80	0.049	1.83	0.52	5.84	21.11	8.32	10.29	19.83
	8	23.74	0.046	1.89	0.44	5.70	22.78	6.24	9.77	22.30
	12	23.32	0.071	2.08	0.56	5.29	22.76	13.35	10.75	23.19
Elephant grass	0	28.84	0.053	1.99	0.73	6.19	27.73	3.25	5.67	18.51
	4	25.45	0.059	2.09	0.49	5.06	24.93	2.94	5.59	18.70
	8	22.86	0.059	2.03	0.39	5.08	22.36	1.77	5.00	15.69
	12	36.02	0.050	2.02	0.68	7.12	34.84	3.52	7.38	25.10
Corn stalk	0	40.42	0.050	1.88	0.78	9.60	38.78	0.88	5.39	16.91
	4	45.77	0.044	1.86	0.77	10.36	43.49	0.93	5.94	19.60
	8	41.32	0.046	1.86	1.79	9.32	39.19	2.55	6.10	20.25
	12	52.40	0.052	2.09	0.93	9.22	50.59	1.30	6.07	26.44
Oil palm leaves	0	15.59	0.061	1.82	0.55	4.96	14.97	3.27	11.78	26.50
	4	13.97	0.064	1.90	0.28	4.21	13.59	2.48	11.21	27.33
	8	15.88	0.059	1.90	0.27	4.48	15.39	2.01	11.13	27.97
	12	14.74	0.063	1.90	0.24	4.41	14.33	1.90	13.69	35.11
Rice straw	0	28.45	0.061	2.06	0.78	5.81	27.78	3.87	8.65	29.23
	4	24.27	0.049	1.84	0.44	6.37	23.37	2.64	11.04	25.03
	8	21.70	0.052	1.78	0.52	6.39	20.96	2.45	9.09	22.29
	12	23.02	0.050	1.82	0.45	6.25	22.27	3.37	10.92	25.62
Sugarcane leaves	0	23.62	0.047	1.80	0.39	6.40	22.76	1.81	8.08	18.69
	4	28.98	0.046	1.83	0.52	7.28	27.81	3.03	10.21	21.81
	8	26.81	0.058	2.00	0.74	5.93	26.04	3.89	7.37	20.86
	12	25.17	0.049	1.85	0.55	6.39	24.30	2.95	8.00	18.81
Pooled SEM ^3^		1.700	0.003	0.045	0.144	0.445	1.546	0.572	0.606	1.289
*p*-value										
Forage or by-product		<0.0001	<0.0001	<0.0001	<0.0001	<0.0001	<0.0001	<0.0001	<0.0001	<0.0001
Seaweed level		0.0034	0.2131	0.0671	0.1412	0.4892	0.0010	0.0021	0.0017	<0.0001
Linear		0.0369	0.2836	0.3444	0.5934	0.3061	0.0210	0.0395	0.0089	0.0068
Quadratic		0.0105	0.9804	0.4574	0.3940	0.3323	0.0058	0.0223	0.0032	0.0008
Forage or by-product × Seaweed level		<0.0001	<0.0001	<0.0001	0.0018	0.0826	<0.0001	<0.0001	<0.0001	<0.0001

^1^ b, asymptotic CH_4_ production (mL g^−1^ DM); c, rate of CH_4_ production (h^−1^); Lag, initial delay before CH_4_ production begins (h); ^2^ TG, total gas. ^3^ SEM, standard error of the mean.

### 3.2 Degradability and volatile fatty acid profile

The degradability and volatile fatty acid (VFA) profiles during *in vitro* rumen fermentation of different substrates with increasing levels of *Eucheuma cottonii* are presented in Tables 5, 6, respectively. Both dry matter digestibility (DMD) and organic matter digestibility (OMD) significantly increased (*p* < 0.0001) with higher levels of seaweed inclusion. Among the substrates, corn stalks exhibited the highest (*p* < 0.0001) DMD and OMD, whereas palm oil leaves and sugarcane tops recorded the lowest DMD and OMD, respectively. In general, diets containing seaweed showed enhanced degradability across the forages. Fermentation profiles revealed that corn stalks resulted in the lowest (*p* < 0.0001) rumen pH, ammonia nitrogen (NH₃-N, mg/g DM), and partitioning factor at 24 h (PF24, mg DMD/mL gas), while showing the highest (*p* < 0.0001) metabolizable energy (ME, MJ/kg DM) and microbial crude protein (MCP, mg/g DM). Conversely, palm oil leaves exhibited the highest rumen pH and PF24 but had the lowest ME and MCP values. Increasing seaweed levels led to a general trend of decreasing ME, increasing MCP, and increasing PF24, with the exception of the 8% seaweed level, where PF24 was significantly lower (*p* < 0.05) than the control. An interaction between substrate type and seaweed level was observed. The highest ME values were recorded in corn stalk and palm oil leaves without seaweed; rice straw, sugarcane tops, and buffel grass with 8% seaweed; and elephant grass with 4% seaweed.

**Table 5:** pH, degradability, and *in vitro* ruminal fermentation profile of different forages and crop by-products supplemented with increasing levels of the *seaweed Eucheuma cottonii.*

**Forage and by-product**	**Seaweed level (%)**	**pH** ^**1**^	**Degradability** ^**2**^		**Ruminal fermentation profile** ^**3**^			
			**DMD**	**OMD**	**NH**_**3**_ **-N**	**ME**	**MCP**	**PF**_**24**_
Buffel grass	0	6.88	72.42	67.33	229.50	6.05	677.26	6.85
	4	6.91	66.89	67.94	238.00	5.59	632.13	7.49
	8	6.92	71.03	65.38	242.25	5.91	665.92	7.05
	12	6.92	74.35	67.89	238.00	5.75	701.64	7.86
Elephant grass	0	6.94	54.06	48.07	212.50	3.88	520.13	12.67
	4	6.95	50.57	46.16	212.50	4.32	481.83	8.63
	8	6.94	51.17	50.83	208.25	4.31	484.52	8.68
	12	6.95	55.25	50.31	208.25	4.09	529.96	11.00
Corn stalk	0	6.72	79.06	79.26	170.00	7.41	709.82	4.39
	4	6.75	86.00	85.27	148.75	7.29	792.65	4.98
	8	6.75	86.73	86.10	170.00	6.73	798.38	5.60
	12	6.79	74.09	77.24	153.00	6.73	671.00	4.78
Oil palm leaves	0	6.92	45.05	48.52	182.75	3.84	430.26	10.56
	4	7.04	42.70	49.01	178.50	3.71	408.88	11.21
	8	7.06	44.65	50.17	136.00	3.78	428.73	11.07
	12	6.92	55.77	58.57	174.25	3.56	543.53	17.31
Rice straw	0	6.88	51.10	65.19	174.25	4.43	483.12	7.55
	4	6.90	65.28	71.41	191.25	4.25	624.30	10.80
	8	6.90	53.40	59.29	216.75	4.50	502.02	7.54
	12	6.89	61.60	67.81	157.25	4.19	588.77	10.45
Sugarcane leaves	0	6.88	49.48	47.68	216.75	4.74	461.32	6.37
	4	6.88	49.99	47.06	178.50	4.58	468.27	6.91
	8	6.88	51.11	47.16	199.75	4.80	475.26	6.34
	12	6.89	51.73	46.96	182.75	4.77	482.32	6.53
Pooled SEM ^4^		0.038	0.623	0.574	8.632	0.081	5.225	0.333
*p*-value								
Forage or by-product		<0.0001	<0.0001	<0.0001	<0.0001	<0.0001	<0.0001	<0.0001
Seaweed level		0.2600	<0.0001	<0.0001	0.0874	0.0001	<0.0001	<0.0001
Linear		0.0604	0.1736	0.2871	0.9643	0.7438	0.0586	0.0002
Quadratic		0.5045	<0.0001	<0.0001	0.0237	0.0001	<0.0001	<0.0001
Forage or by-product × Seaweed level		<0.0001	<0.0001	<0.0001	0.6547	<0.0001	<0.0001	<0.0001

^1^ pH, ruminal pH at 48 h of incubation. ^2^ DMD, dry matter digestibility (%); OMD, organic matter digestibility (%). ^3^ NH_3_-N, is ammonia-N (mg g^−1^ DM); ME, metabolizable energy (MJ kg^−1^ DM); MCP, microbial CP production (mg g^−1^ DM); PF_24_, the partitioning factor at 24 h of incubation (mg DMD mL^−1^ gas). ^4^ SEM, standard error of the mean.

**Table 6:** Total concentration and profile of short-chain fatty acids in different types of forages and crop by-products supplemented with increasing levels of the seaweed *Eucheuma cottonii*.

**Forage and by-product**	**Seaweed level (%)**	**Total conc. SFCA** ^**1**^	**Profile of short-chain fatty acids** ^**2**^						**AA/PA ratio** ^**3**^
			**AA**	**PA**	**Iso-but**	**BA**	**Iso-val**	**Valeric**	
Buffel grass	0	41.92	25.30	11.83	0.43	3.27	0.48	0.62	2.15
	4	35.57	22.08	9.36	0.42	2.74	0.41	0.58	2.36
	8	32.15	19.86	8.44	0.34	2.63	0.39	0.52	2.40
	12	49.65	30.42	12.46	0.70	4.31	0.67	1.10	2.46
Elephant grass	0	30.35	19.92	7.44	0.31	2.17	0.20	0.33	2.68
	4	28.45	18.38	7.11	0.26	2.15	0.21	0.34	2.59
	8	28.68	19.14	6.47	0.37	2.18	0.21	0.32	3.03
	12	28.64	19.01	6.40	0.46	2.23	0.21	0.35	2.98
Corn stalk	0	60.72	35.35	17.01	0.86	5.51	1.29	0.71	2.08
	4	63.55	38.37	16.72	1.06	5.62	0.86	0.94	2.30
	8	57.65	34.72	15.41	0.98	4.77	0.97	0.80	2.25
	12	73.10	44.37	19.03	1.28	6.10	1.38	0.95	2.33
Oil palm leaves	0	25.46	17.74	4.62	0.62	1.92	0.24	0.34	4.02
	4	24.36	15.99	4.28	0.72	2.03	0.84	0.51	3.76
	8	21.60	13.73	4.70	0.75	1.76	0.27	0.40	2.95
	12	29.61	17.96	5.75	0.89	2.60	1.35	1.07	3.14
Rice straw	0	38.74	23.81	9.68	0.88	3.32	0.53	0.54	2.47
	4	33.35	20.20	8.41	0.71	2.85	0.66	0.54	2.41
	8	29.17	18.11	7.13	0.43	2.43	0.59	0.49	2.55
	12	31.97	19.07	7.65	0.67	2.99	0.89	0.70	2.53
Sugarcane leaves	0	30.30	19.52	7.32	0.40	2.43	0.25	0.39	2.67
	4	41.44	24.12	9.88	0.68	5.86	0.33	0.57	2.47
	8	35.30	22.30	8.62	0.61	3.05	0.21	0.52	2.59
	12	33.09	20.83	8.12	0.59	2.84	0.24	0.48	2.58
Pooled SEM ^4^		2.510	1.367	0.714	0.098	0.422	0.141	0.109	0.140
*p*-value									
Forage or by-product		<0.0001	<0.0001	<0.0001	<0.0001	<0.0001	<0.0001	<0.0001	<0.0001
Seaweed level		<0.0001	0.0020	0.0049	0.0049	0.0085	0.0003	<0.0001	0.9225
Linear		<0.0001	0.0020	0.0020	0.5003	0.2617	0.1154	0.5067	0.4963
Quadratic		<0.0001	0.0015	0.0668	0.0033	0.0014	0.0008	0.0001	0.9361
Forage or by-product × Seaweed level		<0.0001	0.0003	0.0037	0.0792	0.0004	0.0056	0.0504	0.0002

^1^ Total conc. SFCA, total concentration of short-chain fatty acids (mmol g^−1^ DM). ^2^ AA, acetic acid (mmol g^−1^ DM); PA, propionic acid (mmol g^−1^ DM); Iso-but, iso-butyric acid (mmol g^−1^ DM); BA, butyric acid (mmol g^−1^ DM); Iso-val, iso-valeric acid (mmol g^−1^ DM); Val, valerate (mmol g^−1^ DM); ^3^ AA/PA, acetic acid/propionic acid ratio. ^4^ SEM, standard error of the mean.

The VFA profile showed that palm oil leaves produced the lowest (*p* < 0.0001) concentrations of total SCFA, acetic acid, propionic acid, butyric acid, and resulted in the highest acetate-to-propionate ratio among all forages during the 48-h incubation. In contrast, corn stalk yielded the highest concentrations of these VFAs, leading to the lowest acetate-to-propionate ratio (Table 6). During digestion, elephant grass had the lowest iso-butyric acid concentration, while corn stalk had the highest, followed by palm oil leaves. Elephant grass also recorded the lowest (*p* < 0.0001) levels of valeric and iso-valeric acids, whereas corn stalk had the highest. A similar trend was observed with seaweed inclusion. Diets containing 8% seaweed showed significantly lower concentrations of acetic acid (*p* = 0.002), propionic acid (*p* = 0.0049), butyric acid (*p* = 0.0085), iso-butyric acid (*p* = 0.0049), and iso-valeric acid (*p* = 0.0003), whereas diets with 12% seaweed had the highest levels of these VFAs. The VFA profile also varied by substrate depending on the seaweed inclusion level. Specifically, total SCFA, acetic, propionic, and butyric acids were highest (*p* < 0.05) in corn stalk, palm oil leaves, and buffel grass with 12% seaweed; sugarcane tops with 4% seaweed; and in elephant grass and rice straw diets without seaweed.

### 3.3 Rumen bacterial abundance and diversity

Diversity estimates of 16S rRNA gene sequencing data from the rumen microbiota of Ongole crossbreed grade cattle are given in Table 7. The results indicate that rice straw without seaweed had slightly more species than rice straw with 8% seaweed inclusion. The higher Shannon index in rice straw without seaweed suggests greater microbial diversity compared to rice straw with 8% seaweed group. Furthermore, the higher Simpson index in straw group indicates a more evenly distributed microbial community, suggesting a lower dominance of specific taxa compared to straw with 8% seaweed group. Chao1 and ACE (Abundance-based Coverage Estimator) which is higher in rice straw without seaweed compared to rice straw with 8% seaweed showed that rice straw only likely had more total richness from the undetected species indicating it contained more diversity than rice straw with 8% seaweed group.

**Table 7:** Diversity estimates based on the 16S rRNA gene of rumen microbiota in rice straw without and with supplementation of 8% seaweed (*Eucheuma cottonii*), after 48 h of *in vitro* fermentation.

Treatment ^1^	Total reads	Observed species	Shannon	Simpson	Chao1	ACE
RS0	172,288	1,655	8.306	0.99	1837.782	1780.118
RS8	189,200	1,607	7.887	0.983	1688.281	1701.254

^1^ RS0, rice straw without seaweed supplementation; RS8, rice straw supplemented with 8% seaweed.

**Figure 4:**
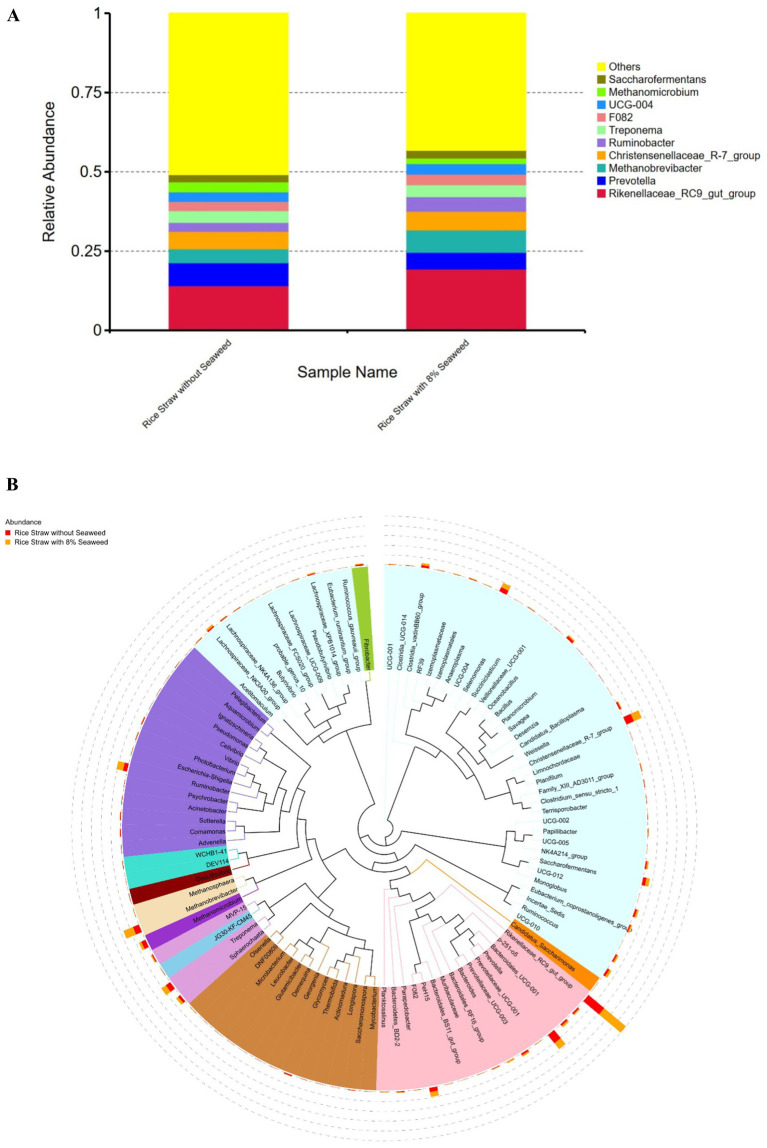
**(A)** Relative abundance of ruminal microbial genera in rice straw, with and without 8% supplementation of the seaweed *Eucheuma cottonii*, after 48 h of *in vitro* ruminal fermentation. **(B)** Phylogenetic tree of ruminal microbiota based on 16S rRNA sequencing in rice straw, with and without 8% supplementation of the seaweed *Eucheuma cottonii*, after 48 h of *in vitro* ruminal fermentation.

**Figure 5:**
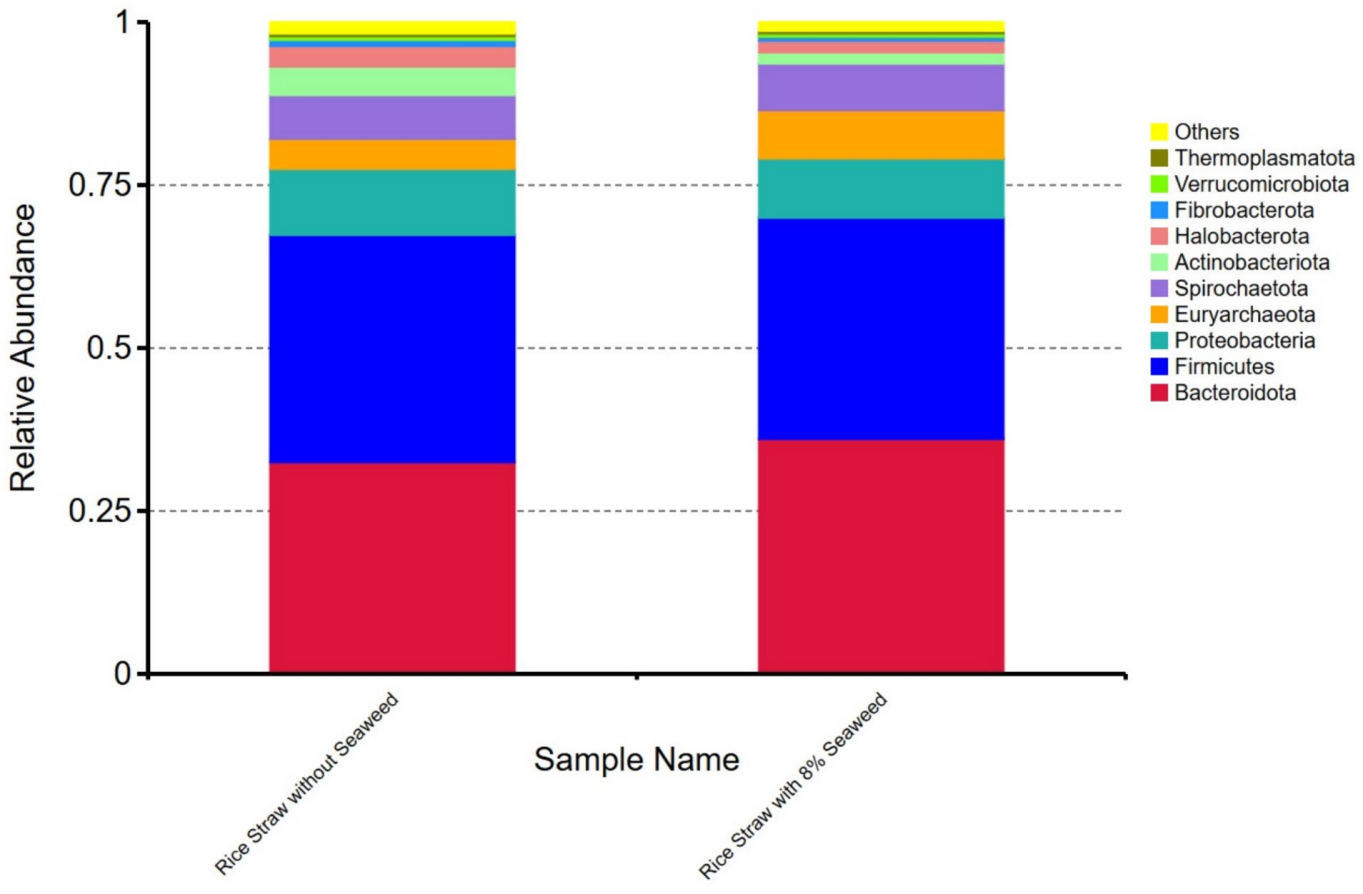
Relative abundance of ruminal microbial phyla in rice straw, with and without supplementation with 8% of the seaweed *Eucheuma cottonii*, after 48 h of *in vitro* ruminal fermentation.

**Figure 6:**
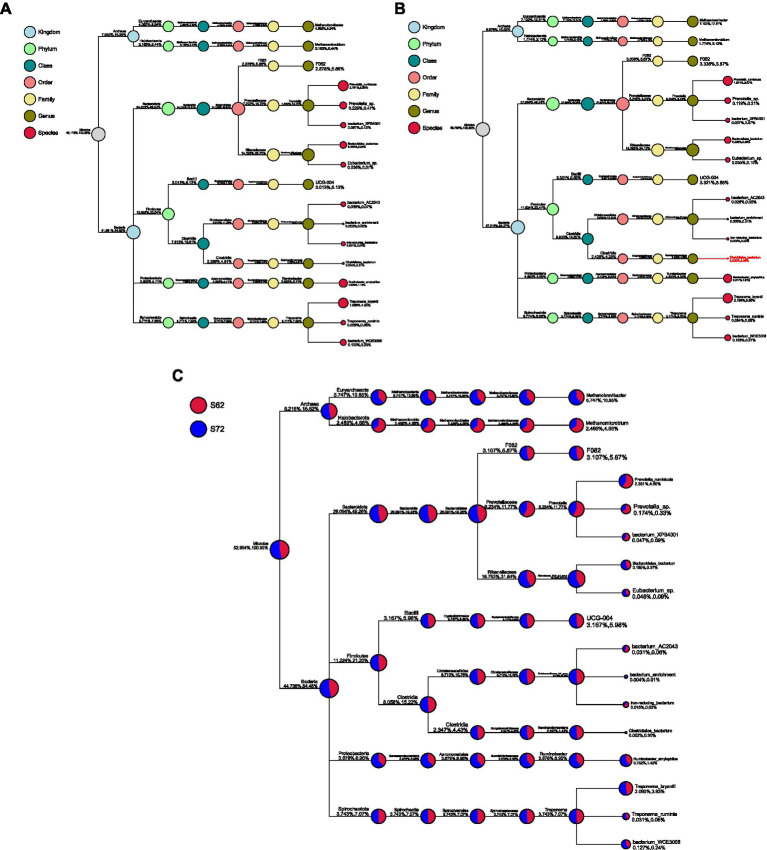
**(A)** Phylogenetic Tree of rumen microbiota based on 16S rRNA sequencing of rice straw with 0% seaweed *Eucheuma cottonii*, after 48 h of *in vitro* ruminal fermentation. **(B)** Phylogenetic Tree of rumen microbiota based on 16S rRNA sequencing of rice straw with 8% seaweed *Eucheuma cottonii*, after 48 h of *in vitro* ruminal fermentation. **(C)** Phylogenetic tree of ruminal microbiota based on 16S rRNA sequencing of rice straw, with and without supplementation of 8% of the seaweed *Eucheuma cottonii*, after 48 h of *in vitro* ruminal fermentation.

Regarding the relative abundance of microbial taxa at genus level (Figures 4A,B), unclassified taxa constituted the largest proportion in rice straw without seaweed, accounting for 50.88%, followed by *Rikenellaceae_RC9_gut_group* (14.12%), *Prevotella* (7.22%), *Methanobrevibacter* (4.39%), and *Methanomicrobium* (3.16%). In contrast, in rice straw with 8% seaweed, unclassified taxa accounted for 43.21%, followed by *Rikenellaceae_RC9_gut_group* (19.38%), *Prevotella* (5.24%), *Methanobrevibacter* (7.10%), and *Methanomicrobium* (1.77%). *Rikenellaceae_RC9_gut_group*, *Methanobrevibacter*, and *Ruminobacter* were more abundant in the rice straw with 8% seaweed group, which also exhibited a lower proportion of unclassified taxa. In contrast, *Prevotella*, *Methanomicrobium*, and unclassified taxa were more dominant in the rice straw group. At the phylum level, the microbial composition of the rice straw group consisted of 32.49% *Bacteroidota*, 34.86% *Firmicutes*, 10.14% *Proteobacteria*, and 0.92% *Fibrobacter*. The rice straw with 8% seaweed group, however, showed a composition of 36.06% *Bacteroidota*, 34.86% *Firmicutes*, and 0.60% *Fibrobacter* (Figure 5). In Figures 6A–C notable differences between rice straw without seaweed group and rice straw with seaweed group were observed at taxonomical level. The combined proportion of *Bacteroidota* and *Firmicutes* was 67.34% in the rice straw without seaweed group and 69.97% in the rice straw with 8% seaweed group. The analysis revealed that bacteria constituted 84.48% of the microbial population, while archaea accounted for 15.52%. Within the bacterial community, *Bacteroidota* (49.28%), *Firmicutes* (21.20%), *Proteobacteria* (6.95%), and *Spirochaetota* (7.07%) were predominant. The *Rikenellaceae_RC9_gut_group* was the dominant genus within Bacteroidota, *Christensenellaceae_R-7_group* within Firmicutes, and *Ruminobacter* within Proteobacteria. *Treponema* was the leading genus in Spirochaetota. Among the archaea, Euryarchaeota was primarily represented by *Methanobrevibacter*, accounting for 10.85% of archaeal sequences, while Halobacterota was dominated by *Methanomicrobium*, comprising 4.66% of archaeal sequences. The supplementation of rice straw with 8% seaweed influences rumen microbial composition, increasing the abundance of specific genera such as *Rikenellaceae_RC9_gut_group*, *Methanobrevibacter*, and *Ruminobacter*, while reducing the proportion of unclassified taxa.

## 4 Discussion

### 4.1 Ruminal total gas production and methane

Crop byproducts and agricultural waste can be effectively incorporated into ruminant diets, as these animals possess a unique ability to digest fibrous crop biomass that monogastric find difficult to process. Gas production may be used as a good indicator of nutrient digestibility, fermentability, and microbial protein production (32, 33). Furthermore, the higher gas production in corn stalks may be due to the combination of higher dry matter (DM) and organic matter (OM) with lower crude fiber (CF), making it easier for rumen microbes to quickly adapt and utilize the soluble components for digestion, perhaps due to the presence of soluble carbohydrates compared to other forages (34). Plant species greatly affected the chemical composition, gas and methane production, ME, and OMD of plants (35).

The ability of rumen microbes to quickly adapt to a substrate and degrade it, influenced by the presence of soluble material, is supported by the shorter lag time. Lag time indicates the period between substrate intake and the first gas production. Additionally, comparing the DM, OM, and CF of sugarcane tops and buffel grass, which were nutritionally closer to corn stalks, the gas produced from corn stalk was 60.82% higher than that of sugarcane tops and 48.52% higher than that of buffel grass. This suggests that, beyond nutritional factors, the rapid accessibility of soluble components in corn stalks supported greater gas production. The lower gas production observed in the oil palm-based diet may be due to its high lignin content and possibly the presence of metabolites such as tannins, which might have inhibited digestion. The availability of readily fermentable carbohydrates can influence rumen degradation, as a high energy supply favors microbial growth and, consequently, the digestion and passage rate of material through the gastrointestinal tract (36). This could explain the pattern observed in gas production per gram of DM incubated, which followed the pattern of asymptotic gas production.

In this study, though not statistically significant, the overall pattern with increasing levels of *E. cottonii* supplementation showed that asymptotic gas production reduced by 8.22% from 0% seaweed to 12% seaweed. This is in contrast to the result reported by Yousaf et al. (15) that *E. cottonii* improves gas productivity *in vitro*. Other studies, such as Munde et al. (37), reported that up to 3% *E. cottonii* did not affect gas production or fermentation parameters, while Sharma et al. (38) found that up to 2% *E. cottonii* increased *in vitro* gas production. The variation in response compared to our study may be due to the higher levels of *E. cottonii* used in our experiment. Compared to Yousaf et al. (15) and Sharma et al. (38), where *E. cottonii* represented just 2% of the diet. In our study, the minimum supplementation level was 4%. This suggests that high levels of *E. cottonii* may be ineffective or even inhibitory to proper rumen fermentation *in vitro*. Looking at the interaction between seaweed inclusion levels and the different forages, there was no clear pattern of asymptotic gas production. However, most of the highest gas production values occurred within the 0–4% *E. cottonii* range across all forages. This suggests that, for the forage samples tested, high levels (above 4%) of *E. cottonii* limit the ability of rumen microbes *in vitro* to effectively utilize and ferment substrates.

Methane production accounts for substantial dietary energy losses, significantly impacting ruminant productivity (39). Additionally, enteric CH₄ emissions from ruminants contribute approximately 17% of global greenhouse gas emissions (40). Although CH₄ from ruminants is biogenic in nature, its short-term environmental impact necessitates efforts to reduce emissions. Lower *in vitro* gas production combined with high methane emission suggests that a greater proportion of the fermented substrate is being converted into methane rather than total gas volume. This could be due to the high lignin content present in palm oil leaves or an increased protozoa population. It is well known that there is a relationship between protozoa and methanogens, which could have led to increased methane production. Less fermentable carbohydrates result in lower CO₂ and H₂ accumulation, reducing total gas output while increasing the proportion of CH₄. The highest level of gas produced in the corn stalk group may be associated with the higher fermentation. However, comparing the proportion of methane at 24 h to total gas produced showed that it had the lowest methane. The seaweed with the lowest methane is 8% seaweed. This is attributed to the presence of sulfated polysaccharides, which inhibit methane formation (41). In their work, King et al. (41) demonstrated that in anaerobic environments, *E. cottonii* promotes CO₂ production rather than methane. Both *in vivo* and *in vitro* studies have shown that *E. cottonii* reduces methane emissions even when included at 1–4% of the diet (15, 16, 42, 43). This methane reduction could be due to the presence of compounds such as bromoform, hydrocolloid carrageenan, and polyphenolic compounds (similar to phlorotannins) in the seaweed, which have anti-methanogenic effects (15).

### 4.2 Ruminal fermentation parameters and degradability

Dry matter digestibility (DMD) of a feed or forage is a key indicator of its nutritional quality and potential to provide energy to animals (34, 44). In addition, organic matter digestibility (OMD) is a measure of available energy and fermentable substrates for ruminants and can be used to assess microbial degradation of substrates in the presence of sufficient ammonia nitrogen (45). In this study, the high levels of DMD and OMD may be attributed to increased rumen microbes such as fungi and fibrolytic bacteria activity (46) suggesting that microbes were able to access soluble nutrients more quickly than with other substrates. It is also possible that corn stalks contain more soluble nutrients and highly degradable components compared to other substrates, or both factors could be contributing or rumen fungi likely played a role in breaking down the complex plant cell wall structure before the microbes could access the soluble sugars (47). The lower DMD and OMD observed in palm oil leaves may be attributed to their fibrous nature or high lignin content, which makes it difficult for microbes to break down and access the nutrients for degradation compared to other substrates. In addition, Santoso et al. (48), Rusli et al. (49), and Arpinaini et al. (50) reported that high fiber content, low soluble carbohydrate levels (approximately 22%), and lignin content (around 19%) contribute to the poor digestibility of palm oil leaves. These factors likely account for the low digestibility observed in this study, especially since the material was used at 100% inclusion without any form of pretreatment. Thus, pretreatment is often necessary before use to enhance the feed value of palm oil leaves by improving their fermentability and nutrient availability. The increased DMD and OMD observed in this study align with the findings of Yousef et al. (15), who reported that *E. cottonii* improved digestibility. However, the increased digestibility did not correspond with increased gas production. This could suggest that rather than being converted into gas, digestion may have resulted in the production of other byproducts, such as microbial crude protein, which is more beneficial to ruminants than gas production, particularly if the gas produced included more CO₂.

Gas production is directly proportional to short-chain fatty acids (SCFAs), meaning that higher gas production corresponds to higher SCFA levels (45, 51). SCFA levels indicate energy availability and can contribute up to 80% of an animal’s daily energy requirements (52). They are also directly proportional to ME and OMD (53). In this study, the corn stalk substrate produced the highest levels of SCFAs, including total SCFA, acetic, propionic, butyric, iso-butyric, valeric, and iso-valeric acids, resulting in the lowest acetate-to-propionate (A:P) ratio. The increase in SCFA production may be attributed to more effective fermentation of the substrate, leading to greater organic acid production as a natural byproduct of microbial activity. In contrast, palm oil leaves had the lowest SCFAs concentrations (total SCFA, acetic, propionic, and butyric acids) among the fermented forages, corresponding with lower fermentation levels, as reflected in the gas production at 48 h. SCFAs (acetic, propionic, and butyric acids, as well as total SCFA) showed a decrease at 4 and 8% seaweed inclusion compared to the control but then increased at 12%, surpassing even the control diet. The reason for this unexpected increase in SCFA at 12% seaweed inclusion, while lower doses reduced it, remains unclear. However, when examining branched-chain volatile fatty acids (BCVFAs), such as valeric, isobutyric, and isovaleric acids, there was a consistent increase with rising seaweed levels. These BCVFAs play a crucial role in promoting microbial protein synthesis and supporting the growth of cellulolytic (fiber-digesting) bacteria (54, 55).

BCVFAs help mitigate the negative effects of low-quality fiber by enhancing microbial efficiency and digestion. The shift toward BCVFAs and possibly increased microbial crude protein (MCP) production suggests that, due to the low-quality fiber content of the diet used in our experiment, fermentation may have favored alternative microbial pathways (55). Wang et al. (55) found that diet containing BCVFA enhances celluloytic bacteria population and fiber degradability. This is further supported by microbial profiling, which showed that diets containing seaweed (particularly with rice straw) resulted in an improved proportion of *Fibrobacter* species, cellulolytic bacteria responsible for fiber digestion, compared to rice straw without seaweed [Yen et al. (56); Figure 4A].

Rumen pH is a key parameter used to assess the acidity or alkalinity of rumen fermentation. It can be influenced by diet, which in turn affects CO₂ dynamics in the rumen fluid. Diets that promote high CO₂ retention can lead to increased dissolved CO₂ (*d*CO₂) concentrations, resulting in a concomitant decline in rumen pH (57). In this study, rumen pH values ranged between 5.5 and 7.5, which is within the expected range for high-forage diets (58–60). The lowest rumen pH values, though still within the normal range, were observed in forages without seaweed supplementation.

The forage (buffel grass) with the highest NH₃-N concentration was likely fresh before processing. Its proximate composition revealed a higher protein content than other forages, which could explain the elevated ammonia nitrogen levels. Interestingly, NH₃-N concentrations decreased with increasing seaweed inclusion, while microbial crude protein (MCP) synthesis increased. This suggests that seaweed supplementation enhanced microbial protein synthesis rather than simply contributing to gas production, providing a more valuable nutritional outcome. The high MCP levels observed in the corn stalk-based diet may be attributed to the presence of soluble sugars or readily available substrates, which, in combination with ammonia nitrogen, created an optimal carbon-to-nitrogen (C:N) balance for microbial proliferation. Studies have shown that matching the release rates of ammonia from non-protein nitrogen (NPN) sources with the fermentation rates of carbohydrates enhances microbial protein synthesis (61).

Similarly, the high ME values observed for corn stalks can be attributed to efficient fermentation. Partitioning factor at 24 h (PF_24_) is an indicator of the amount of digestible dry matter (DMD) degraded per mL of gas produced after 24 h of incubation. A higher PF_24_ value indicates that more substrate is utilized for microbial protein synthesis rather than lost as fermentation gases (CO₂, CH₄), whereas a lower PF_24_ suggests greater fermentation into gas and less incorporation into microbial biomass (44). Corn stalks had the lowest PF_24_, whereas oil palm leaves had the highest, indicating that corn stalk fermentation favored microbial protein synthesis. Additionally, the effect of seaweed supplementation showed an increasing PF_24_ trend with higher seaweed inclusion levels, which was reflected in the increased DMD. The interaction between seaweed and forages indicated that, although the trend varied by forage type, seaweed supplementation consistently resulted in the highest MCP levels and PF_24_ values compared to diets without supplementation.

### 4.3 Rumen bacteria community of two diet

The Shannon and Simpson indices of microbial diversity in the *in vitro* diet samples containing rice straw, showed that rice straw without seaweed supplementation had higher species richness, greater diversity, and an even microbial distribution than the diet containing seaweed. A possible reason for the higher species richness in rice straw without seaweed is that introducing seaweed may have reduced the abundance of some microbes while promoting the growth of others that can handle seaweed components alongside rice straw. During digestion, the substrates in rice straw alone were relatively uniform. Rumen microbiome consists of a network of microbes capable of digesting a wide range of ingredients. The dominance of specific microbes at any given time is often influenced by their preference for particular feed structures and substrates (62). Looking at microbial relative abundance at the genus level, there was a shift from the dominance of unclassified taxa in rice straw without seaweed to more defined microbial populations in rice straw with seaweed (Figures 3A,B). This shift was driven by an increase in *Rikenellaceae RC9 gut group*, *Methanobrevibacter*, and *Ruminobacter*, with a reduction in *Methanomicrobium*. A high proportion of this group supports previous findings that *Bacteroidetes* dominate in hay-based diets, while *Firmicutes* tend to be more dominant in high-grain diets (62).

Rice straw with seaweed contained more *Bacteroidiota* compared to *Firmicutes*. The observed microbial shift, characterized by an increase in *Rikenellaceae RC9 gut group, Methanobrevibacter,* and *Ruminobacter*, suggests that these microbes were better adapted to the seaweed-containing diet, allowing them to dominate while reducing the proportion of unclassified taxa. Although shifts were observed, *Bacteroidota* and *Firmicutes* remained the predominant phyla, comprising between 67.35 and 69.98% of the microbial community (Figures 6A–C). This proportion is lower than the 80% or more reported by Huang et al. (63) and Faniyi et al. (62). Nevertheless, the core microbial groups, *Bacteroidetes, Firmicutes, Proteobacteria, Fibrobacteres, and Spirochaetes*, were still detected, consistent with findings from their studies. One possible explanation for this difference is the source of the rumen liquor. While most of the studies compared in Faniyi et al. (62) and Huang et al. (63) were often *Bos taurus*, The cattle used in this study were Ongole crossbred cattle (Indian Ongole cattle X native Indonesian cattle) However, in Aprilia (64), who also used Ongole crossbred cattle, the dominant phyla were *Bacteroidetes* (69%), *Proteobacteria* (24%), and *Firmicutes* (4%), with *Psychrobacter* and *Prevotella* being the most abundant genera. Compared to the studies mentioned earlier, the lower proportions of *Bacteroidota* and *Firmicutes* in Aprilia study suggest that cattle breed played a role in shaping the rumen microbiota. This indicates that Ongole crossbred cattle have relatively lower *Bacteroidota* and *Firmicutes* levels. Furthermore, the diversity at the genus level (Figures 4A,B) and the more evenly distributed phylum-level composition in our study, compared to the study of Aprilia (64), suggest that diet influenced microbial diversity. This shows how dietary factors can affect microbial diversity, contributing to a more comprehensive understanding of the rumen microbiota in local cattle breeds in Southeast Asia. The global rumen microbes study by Henderson et al. (46) did not include microbial data from Southeast Asia including Indonesia. However, our findings on *Methanomicrobium* showed a range of 3.12–6.44%, with an average of 4.66%, which is lower than the >5% reported for the archaeal community of cattle from Australia, Brazil, China, North America, and South Africa, as well as South African sheep.

Henderson et al. (46) and Petri et al. (65) have shown that while the rumen has a core microbial community, its composition can shift significantly based on dietary sources. This was evident in this study, where both treatments had the same microbial components but differed in relative abundance due to the presence or absence of *E. cottonii*. This shift may also explain the lower diversity in the rice straw + seaweed group, as fewer microbes could tolerate the seaweed. *Ruminobacter* is a proteolytic bacterium in the rumen, playing a crucial role in fiber digestion and nitrogen metabolism. It contributes to ammonia (NH₃) production, which is essential for microbial protein synthesis. Similarly, the *Rikenellaceae RC9 gut group* is involved in fiber and polysaccharide degradation. The increase in *Rikenellaceae RC9 gut group* may be linked to the presence of polysaccharides in seaweed, which provided a substrate for their proliferation.

*Methanobrevibacter* and *Methanobacterium* are key players in methane production, using hydrogen and carbon dioxide as substrates for methanogenesis (66). In our study, *Methanobrevibacter* increased while *Methanobacterium* decreased in the rice straw + seaweed group. However, despite the increase in *Methanobrevibacter*, methane production still decreased. This suggests that although *Methanobrevibacter* numbers increased, other microbes interacting with them such as protozoa, which play a role in interspecies hydrogen transfer were reduced. Widiawati and Hikmawan (16) reported that *E. cottonii* reduces protozoa and methane production. Since *Methanobrevibacter* makes up over 70% of rumen methanogens (67), the increase in its population and yet decline in methane could be attributed to a decline in protozoa populations (15), due to the relationship between methanogens and protozoa population for methane production (68). Another possible explanation is that sulfate polysaccharides like carrageenan in this seaweed may have consumed some of the hydrogen that would otherwise have been used for methanogenesis (41). The higher gas production in the rice straw without seaweed group compared to the rice straw + seaweed group may be linked to the greater presence of unclassified taxa and the overall higher microbial diversity. A more diverse and evenly distributed microbial community may have had a better ability to digest the sample and produce more SCFAs.

## 5 Conclusion

Cornstalk was the most effective forage among the tested crop by-products, demonstrating the highest gas production, DMD, OMD, SCFA concentrations, ME, and MCP, and the lowest methane percentage. This indicates its high fermentability and nutritional value. Seaweed inclusion influenced fermentation parameters: while gas production, methane, and NH₃-N, decreased with higher *E. cottonii* levels, MCP, DMD, and OMD improved, suggesting enhanced protein synthesis and fiber digestibility. From a microbial standpoint, rice straw + 8% seaweed increased fiber-digesting bacteria. Therefore, for practical application, cornstalks without seaweed are optimal. For areas where cornstalk is unavailable, sugarcane tops with 4% *E. cottonii* offer a viable alternative among crop-by-product. These combinations can enhance ruminant nutrition and potentially reduce enteric methane emissions.

## Data Availability

The data presented in the study are deposited in the NCBI repository, accession number PRJNA1311784 (https://www.ncbi.nlm.nih.gov/sra/PRJNA1311784).
